# Quantitative Phosphoproteomic Comparison of Lens Proteins in Highly Myopic Cataract and Age-Related Cataract

**DOI:** 10.1155/2021/6668845

**Published:** 2021-05-10

**Authors:** Shaohua Zhang, Keke Zhang, Wenwen He, Yi Lu, Xiangjia Zhu

**Affiliations:** ^1^Eye Institute and Department of Ophthalmology, Eye & ENT Hospital of Fudan University, Shanghai 200031, China; ^2^NHC Key Laboratory of Myopia (Fudan University), Key Laboratory of Myopia, Chinese Academy of Medical Sciences, Shanghai 200031, China; ^3^Shanghai Key Laboratory of Visual Impairment and Restoration, Shanghai 200031, China

## Abstract

**Purpose:**

To investigate and compare the lens phosphoproteomes in patients with highly myopic cataract (HMC) or age-related cataract (ARC).

**Methods:**

In this study, we undertook a comparative phosphoproteome analysis of the lenses from patients with HMC or ARC. Intact lenses from ARC and HMC patients were separated into the cortex and nucleus. After protein digestion, the phosphopeptides were quantitatively analyzed with TiO_2_ enrichment and liquid chromatography-mass spectrometry. The potential functions of different phosphopeptides were assessed by Gene Ontology (GO) enrichment analysis and Kyoto Encyclopedia of Genes and Genomes (KEGG) pathway enrichment analysis.

**Results:**

In total, 522 phosphorylation sites in 164 phosphoproteins were identified. The number of phosphorylation sites was significantly higher in the cortex than in the nucleus, in both ARC and HMC lenses. The differentially phosphorylated peptides in the lens cortex and nucleus in HMC eyes were significantly involved in the glutathione metabolism pathway. The KEGG pathway enrichment analysis indicated that the differences in phosphosignaling mediators between the ARC and HMC lenses were associated with glycolysis and the level of phosphorylated phosphoglycerate kinase 1 was lower in HMC lenses than in ARC lenses.

**Conclusions:**

We provide an overview of the differential phosphoproteomes of HMC and ARC lenses that can be used to clarify the molecular mechanisms underlying their different phenotypes.

## 1. Introduction

Cataract is an age-related degenerative disease and the principal cause of blindness worldwide [1]. It frequently accompanies other eye diseases, such as high myopia, glaucoma, uveitis, and trauma, all of which displaying clinical processes distinct from those of age-related cataract (ARC). Highly myopic cataract (HMC) is more prevalent in Asia than in other regions [2–4]. Compared with ARC, HMC is characterized by earlier onset and cataract with greater nuclear sclerosis and rapid progression [5, 6], which implies that there are unique pathological processes involved in the development of each type of cataract. However, the underlying molecular differences between ARC and HMC remain unclear.

Lens proteins are some of the most long-lived proteins in the body and are the targets of numerous posttranslational modifications [7]. These modifications, especially phosphorylation, are implicated in the regulation of protein solubility and activities [8]. Previous studies demonstrated the differential expression of phosphorylated proteins in normal and cataractous lenses and suggested that protein phosphorylation affects the occurrence and development of cataract [9]. Given the vital role of phosphorylation in protein denaturation and the intriguing clinical differences between HMC and ARC, the differential phosphoproteomes in these two conditions warrant investigation. Differences in the phosphorylation of lens proteins could help distinguish the phenotypes of HMC and ARC lenses.

In this first comparative study of the differential phosphorylation status of HMC and ARC lenses, we quantified the phosphoproteomes of the lens cortex and nucleus separately. This was followed by Gene Ontology (GO) functional analysis and Kyoto Encyclopedia of Genes and Genomes (KEGG) pathway enrichment analysis of the differentially phosphorylated proteins to determine the implications of phosphorylation to the unique phenotype of HMC.

## 2. Materials and Methods

**Figure 1:**
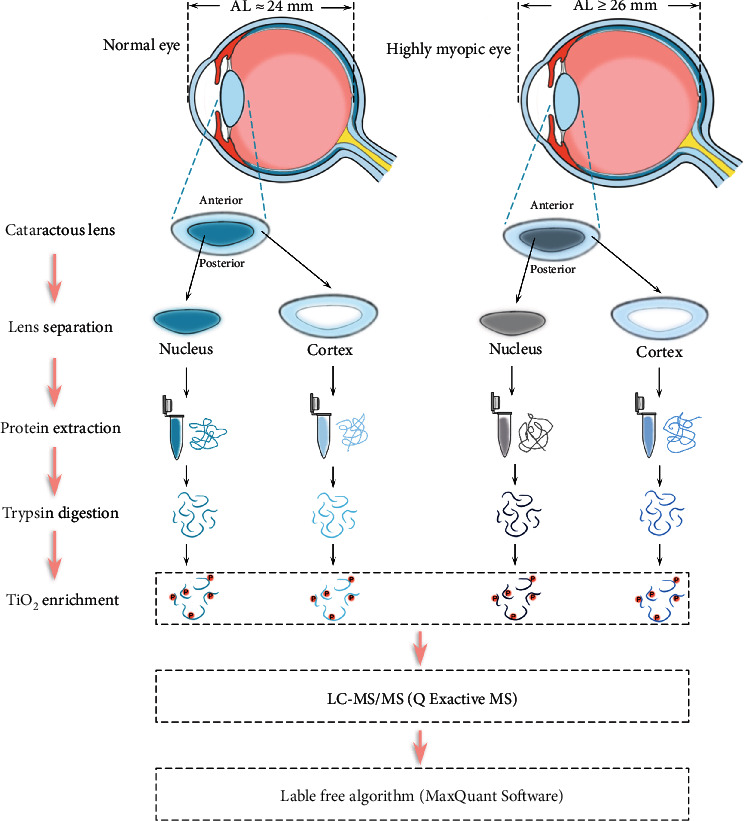
Workflow of the experiments.

In this study, we analyzed the phosphoproteomes of HMC and ARC lenses to detect differences between HMC and ARC. The workflow of this study is shown in Figure 1.

### 2.1. Tissue Collection

In total, three HMC lenses and three age-matched ARC lenses were collected from patients, who provided informed consent before they underwent extracapsular cataract extraction surgery at the Eye and Ear, Nose, and Throat Hospital, Fudan University, Shanghai, China. The research strictly adhered to the tenets of the Declaration of Helsinki and was approved by the Ethics Committee of the Eye and ENT Hospital, Fudan University. Patients with an axial length of ≥26 mm were diagnosed with high myopia. In all lenses, the nucleus and cortex were separated by coring through the visual axis with a 4.5 mm diameter trephine. The lens tissues were divided into four groups: ARC lens cortex (ARC-C), ARC lens nucleus (ARC-N), HMC lens cortex (HMC-C), and HMC lens nucleus (HMC-N).

### 2.2. Sample Preparation

Each tissue was lysed with 200 *μ*L of lysis buffer (4% sodium dodecyl sulfate, 100 mM dithiothreitol, 150 mM Tris-HCl, pH 8.0), disrupted with agitation using a homogenizer, and boiled for 5 min. The samples were ultrasonicated and boiled again for another 5 min. Insoluble material was removed by centrifugation at 12,000 g for 15 min. The supernatant was then collected for protein digestion. The protein concentrations were quantified with a BCA Protein Kit (Bio-Rad, Shanghai, China).

### 2.3. Protein Digestion

We digested the proteins in 250 *μ*g of each sample with the filter-aided sample preparation procedure described by Wisniewski et al. [10]. Briefly, 200 *μ*L of uric acid (UA) buffer (8 M urea, 150 mM Tris-HCl, pH 8.0) was used to remove the detergent, dithiothreitol, and other low-molecular weight components with repeated ultrafiltration. To block the reduction of cysteine residues, 100 *μ*L of 0.05 M iodoacetamide in UA buffer was added and the samples were incubated for 20 min in the dark. The filter was washed three times with 100 *μ*L of UA buffer and then twice with 100 *μ*L of 25 mM NH_4_HCO_3_. The protein suspension was then digested with 3 *μ*g of trypsin in 40 *μ*L of 25 mM NH_4_HCO_3_. The mixture was incubated at 37°C overnight, and the resulting peptides were collected as the filtrate.

### 2.4. Enrichment of Phosphorylated Peptides with TiO_2_ Beads

The phosphopeptides were captured according to the TiO_2_ protocol [11], adapted for label-free quantitative proteomics. The peptides were concentrated with a vacuum concentrator and resuspended in 500 *μ*L of loading buffer (2% glutamic acid, 65% acetonitrile (ACN), and 2% trifluoroacetic acid (TFA)). The TiO_2_ beads were added and then agitated for 40 min and centrifuged for 1 min at 5,000 × *g*. The supernatant was mixed with another TiO_2_ bead, resulting in the second beads which were collected as before. The beads were washed sequentially with 50 *μ*L of washing buffer I (30% ACN, 3% TFA) and 50 *μ*L of washing buffer II (80% ACN, 0.3% TFA) three times to remove the remaining unabsorbed material. The phosphopeptides were finally eluted with 50 *μ*L of elution buffer (40% ACN, 15% NH_4_OH) [12]. The eluates were lyophilized for further analysis.

### 2.5. Liquid Chromatography- (LC-) Electrospray Ionization Tandem Mass Spectrometry (MS/MS) Analysis with Q Exactive™

The peptides from each sample were desalted with a C18 Cartridge (Empore™ SPE Cartridges C18 (standard density), bed I.D. 7 mm, volume 3 mL; Sigma), concentrated with vacuum centrifugation, and equilibrated with 40 *μ*L of 0.1% (*v*/*v*) TFA. MS experiments were performed on a Q Exactive mass spectrometer coupled to an Easy nLC™ liquid chromatography (Thermo Fisher Scientific). The phosphopeptide extract (5 *μ*g) was injected onto a C18 reversed-phase column (Thermo Scientific EASY-Spray™ Column, 10 cm long, 75 *μ*m I.D., 3 *μ*m resin) in buffer A (2% ACN, 0.1% formic acid) and separated with a linear gradient of buffer B (80% ACN, 0.1% formic acid), at a flow rate of 250 nL/min over 60 min. The most abundant precursor ions from the survey scan (300–1800 m*/z*) for higher-energy C trap dissociation (HCD) fragmentation. The target value determination was based on predictive automatic gain control. The duration of dynamic exclusion was 25 s. Survey scans were acquired at a resolution of 70,000 at 200 *m*/*z*, and the resolution of the HCD spectra was set to 17,500 at 200 *m*/*z*. The normalized collision energy was 30 eV, and the underfill ratio, which specifies the minimum percentage of the target value likely to be reached at the maximum fill time, was defined as 0.1%. All MS experiments were performed in triplicate for each sample.

### 2.6. Sequence Database Searches and Data Analysis

All the raw data were identified with the MaxQuant software (version 1.3.0.5.) and screened against the UniProt human database, containing a total of 156,914 entries. The datasets were searched with a mass tolerance of 6 ppm. The search followed the enzymatic cleavage rule: trypsin/P, allowing two missed cleavages; tolerance on a mass measurement of 20 ppm; fixed modification; and carbamidomethylation of cysteines. Protein N-terminal acetylation and methionine oxidation were defined as variable modifications. The cutoff for the global false discovery rate for peptide and protein identification was set to 0.01. Label-free quantification was performed with MaxQuant, as previously described [13]. Protein abundance was calculated based on the normalized spectral protein intensity (label-free quantification (LFQ) intensity). In the quantitative comparison of groups, a protein site was included if it was identified in at least 50% of samples in at least one (HMC or ARC) patient cohort.

### 2.7. Bioinformatics

The GO program Blast2GO was used to annotate the differentially phosphorylated proteins and to create histograms of the GO annotations, including cell components, biological processes, and molecular functions. The KEGG database (KEGG; http://www.genome.jp/kegg/) was used for pathway annotation. The GO terms and KEGG pathways with computed *p* values < 0.05 were considered significantly enriched.

### 2.8. Statistical Analysis

To identify quantitative differences in the phosphorylation states between each group, the degree of phosphorylation at the same site was estimated as the difference ratio and a ratio of >2 was considered to indicate overabundant phosphorylation. Conversely, a reduction in phosphorylation < 0.5-fold was considered to indicate less abundant phosphorylation [14, 15]. Statistical significance was determined with a *t*-test. A *p* value < 0.05 was considered statistically significant.

## 3. Results

### 3.1. Phosphoproteome Identification in HMC and ARC Lenses

**Figure 2:**
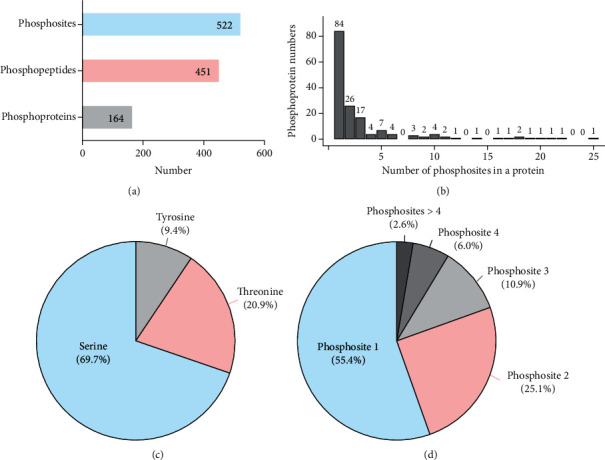
Summary of the phosphoproteomic results. (a). Numbers of identified phosphosites, phosphopeptides, and phosphoproteins. (b). Distribution of phosphorylation sites corresponding to phosphorylated proteins. (c). Proportions of phosphosites with phosphorylation of serine (blue), threonine (pink), and tyrosine (gray). (d). Distribution of the number of phosphorylation sites on each phosphorylated peptide. Each segment represents 1, 2, 3, 4, or >4 phosphosites and corresponds in size to the percentage of phosphopeptides in which this number occurs.

The clinical information for the lens samples is given in Table S1. In this study, we identified 451 unique phosphopeptides in 164 phosphoproteins from the HMC and ARC lens samples (Table S2). Among the 164 phosphoproteins, 84 contained a single phosphorylation site, 26 contained two phosphorylation sites, and 17 contained three phosphorylation sites (Figure 2(b)). The 522 phosphorylation sites identified included 364 on serine (S), 109 on threonine (T), and 49 on tyrosine (Y) accounting for 69.7%, 20.8%, and 9.4% of the total sites, respectively (Figure 2(c)). Among the 451 phosphopeptides, 250 (55.4%), 113 (25.1%), and 49 (10.9%) had one, two, and three phosphorylation sites, respectively. The other 39 phosphopeptides had more than three phosphorylation sites (Figure 2(d)).

### 3.2. Identification of the Differential Phosphopeptides

**Figure 3:**
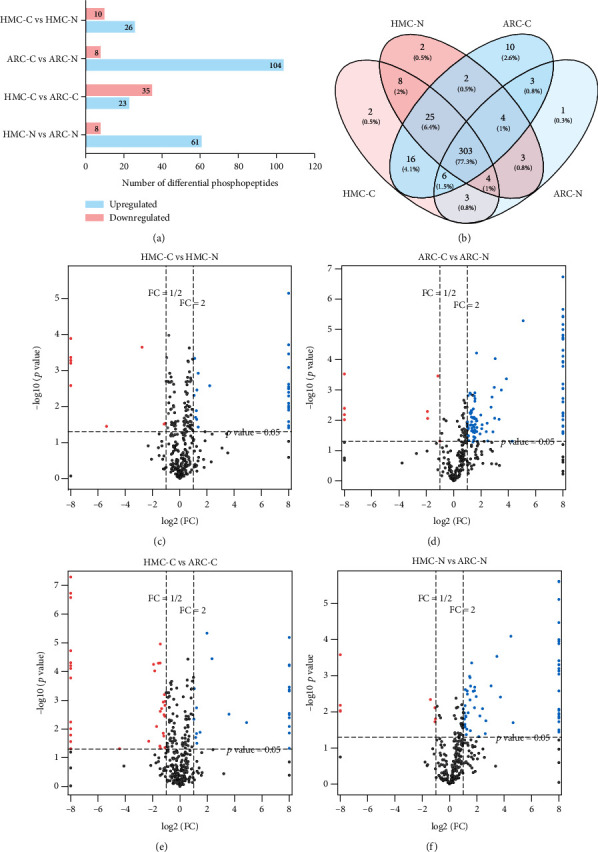
(a) Number of phosphopeptides differing by >2-fold between the groups. (b) Venn diagram of the numbers of phosphopeptides in HMC-C, HMC-N, ARC-C, and ARC-N. (c–f) Volcano plots for the comparisons HMC-C and HMC-N (c), ARC-C and ARC-N (d), HMC-C and ARC-C (e), and HMC-N and ARC-N (f). Phosphosites that are significantly increased or reduced, with a fold-change of >2-fold and *p* < 0.05, are shown as blue and pink circles, respectively. Phosphosites that do not differ significantly are shown as gray circles.

To investigate the quantitative differences in the phosphorylation status of the four groups (ARC-C, ARC-N, HMC-C, and HMC-N), a two-fold change was used as the cutoff to screen for differentially phosphorylated proteins. The level of phosphorylation was higher in the cortex than the nucleus, in both the HMC and ARC lenses. Twenty-six phosphopeptides were hyperphosphorylated in the HMC-C, whereas 10 were underphosphorylated compared with the HMC-N. In the ARC lenses, we detected 104 more abundant phosphopeptides and eight less abundant phosphopeptides in the cortex relative to the nucleus. A comparison of the HMC and ARC lenses revealed that the level of phosphorylation was higher in the HMC-N group than in the ARC-N group. We identified 58 phosphopeptides that were significantly altered in the HMC-C group: 23 were more abundant and 35 were less abundant than in the ARC-C group (Figures 3(a) and 3(c)–3(e)). Among the phosphosites identified, 303 (77.3%) were shared by all four groups (Figure 3(b)).

Seventeen phosphosites were exclusively detected in HMC-C, but not in HMC-N, that included cytoskeletal proteins, oxidoreductases, and binding proteins (Table 1). The phosphorylation status of *S*-formylglutathione hydrolase and glyceraldehyde-3-phosphate dehydrogenase was greater in the HMC-C than in the HMC-N. Meanwhile, in comparisons of ARC-C and ARC-N, a total of 33 phosphosites were exclusively detected in ARC-C and the phosphorylation degree of *β*-crystallin A3 (phosphorylation at site Y36) was much higher in ARC-C, with a different ratio of 34.04. Other significantly overabundant phosphosites were also detected in phakinin, *β*-crystallin B, and filensin (*p* < 0.05) (Table 2).

Twelve and 14 phosphosites were exclusively detected in the HMC-C and ARC-C, respectively, when comparing these groups. A protein with a high degree of phosphorylation was *γ*-crystallin D, which was phosphorylated at Y29 (Table 3). Phosphorylation of the lens cytoskeletal proteins, phakinin and filensin, was significantly lower in the HMC-C than in the ARC-C. In the HMC-N and ARC-N, the predominant differentially expressed phosphosites were found in the *α*-crystallin B chain, at S21, T170, and S76.  Table 4 shows that the proteins with the most abundant phosphorylation included crystallins and structural proteins, particularly *β*-crystallin B1 and filensin.

### 3.3. Gene Ontology and KEGG Pathway Enrichment Analyses

**Figure 4:**
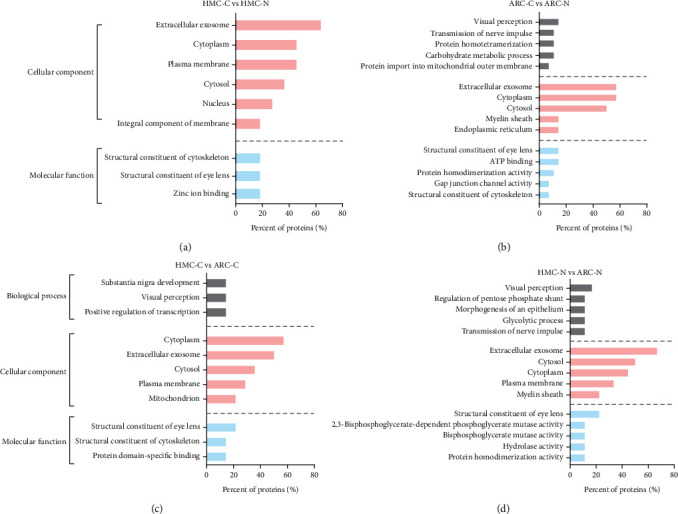
Gene Ontology analysis of differentially phosphorylated proteins for the following comparisons: (a) HMC-C vs HMC-N, (b) ARC-C vs ARC-N, (c) HMC-C vs ARC-C, and (d) HMC-N vs ARC-N. HMC-C: highly myopic cataract lens cortex; HMC-N: highly myopic cataract lens nucleus; ARC-C: age-related cataract lens cortex; ARC-N: age-related cataract lens nucleus.

The potential functions of the phosphoproteins differentially expressed in the different groups were examined by GO analysis. The GO analysis showed that the proteins differentially phosphorylated in the HMC-C and HMC-N were enriched for proteins involved in the cellular compartment, especially in the extracellular exosome, cytoplasm, and plasma. The molecular functions of these proteins were mainly related to the structure of the lens (Figure 4(a)). The GO analysis showed that the differentially phosphorylated phosphopeptides in the ARC lenses were enriched for visual perception, nerve impulse transmission, and protein homodimerization. The differentially phosphorylated phosphoproteins also included cell components, mostly related to the extracellular exosome and cytoplasm, that are involved in the structure of the lens and ATP binding.

When the HMC and ARC groups were compared, the differentially phosphorylated proteins were functionally related to the structure of the lens or were crucial for visual perception in the cortical and nuclear regions. Concerning cellular component, the differentially expressed phosphoproteins in the HMC-C and ARC-C were mainly cytoplasmic proteins. However, the differentially phosphorylated proteins in the nuclear regions of the HMC and ARC groups (HMC-N and ARC-N, respectively) were predominantly extracellular exosome proteins (Figures 4(c) and 4(d)).

**Figure 5:**
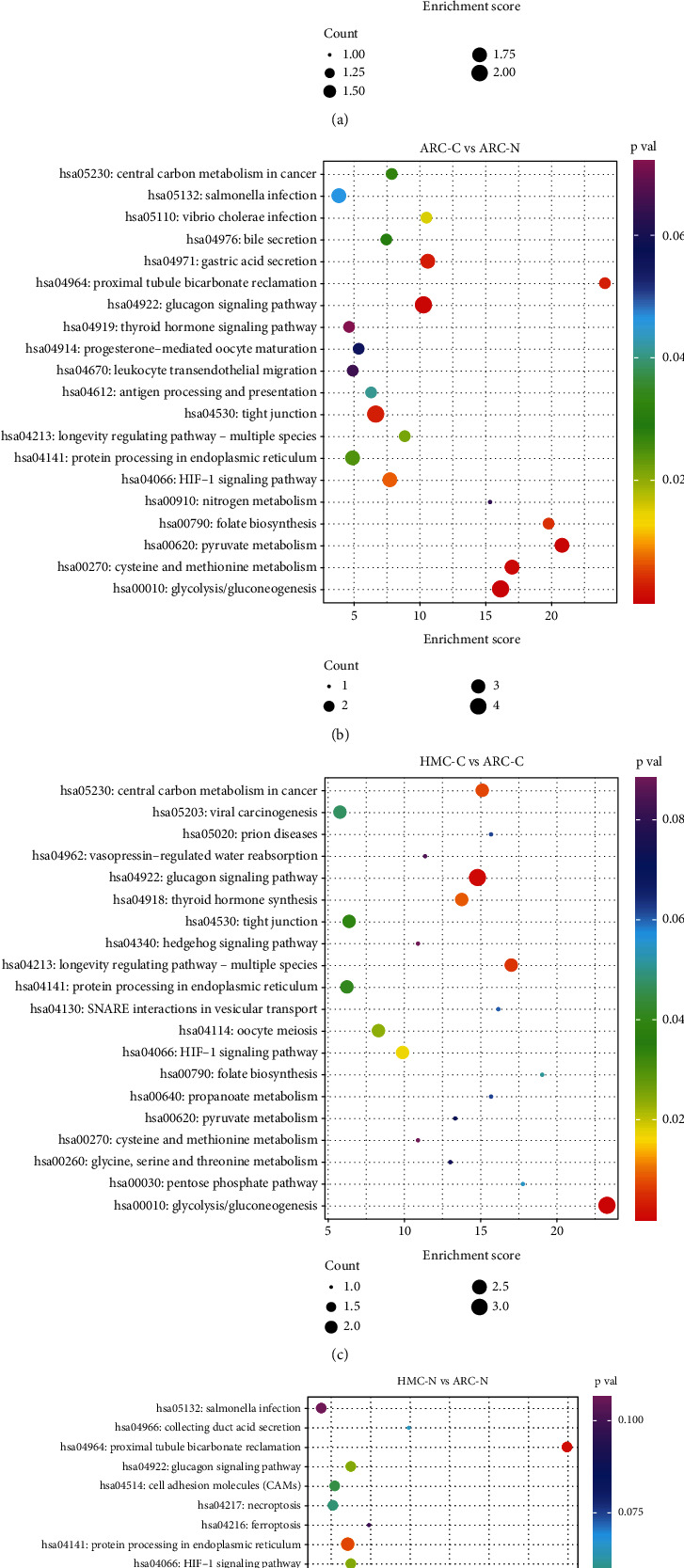
KEGG pathway analysis of differentially expressed proteins. The 20 most enriched terms (*p* < 0.05) are shown for the following comparisons (a) HMC-C vs HMC-N, (b) ARC-C vs ARC-N, (c) HMC-C vs ARC-C, and (d) HMC-N vs ARC-N. HMC-C: highly myopic cataract lens cortex; HMC-N: highly myopic cataract lens nucleus; ARC-C: age-related cataract lens cortex; ARC-N: age-related cataract lens nucleus.

We also performed KEGG pathway enrichment analysis to identify the biological pathways associated with the differentially phosphorylated proteins. The 20 most abundant enrichment terms with *p* < 0.05 are shown in Figure 5. When the cortex and nucleus of HMC were compared, the most significantly enriched pathway was glutathione metabolism. However, when the HMC and ARC lenses were compared, glycolytic enzymes were most frequently differentially expressed.

**Figure 6:**
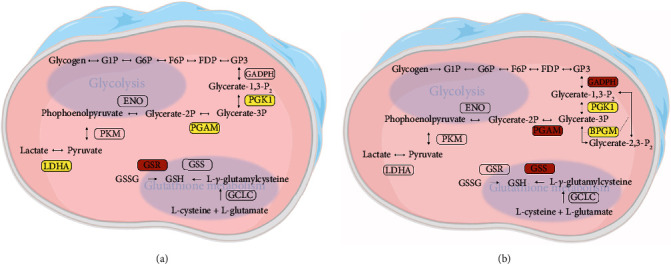
Important pathways associated with the differentially phosphorylated proteins. Differentially phosphorylated proteins were enriched in the glycolysis and glutathione metabolism pathways in the cortex (a) and nucleus (b) of the HMC and ARC lenses. Proteins written in black with a red background are hyperphosphorylated in HMC, whereas those written black with a yellow background are hyperphosphorylated in ARC.

As shown in Figure 6, when we compared the HMC and ARC lenses, the differentially phosphorylated proteins were enriched in the glycolysis and glutathione metabolism pathways. The key glycolytic enzyme, phosphoglycerate kinase 1 (PGK1), was the least phosphorylated protein in the HMC lenses. However, glutathione synthetase (GSS) and glutathione-disulfide reductase (GSR), the key enzymes in glutathione synthesis, were hyperphosphorylated in HMC.

## 4. Discussion

To improve the efficiency of identifying the phosphorylation sites in this study, we used TiO_2_ enrichment combined with LC-MS/MS. For the first time, two parts of the lens with known histological differences, the lens cortex and nucleus, were compared separately. Proteomic differences were detected in the different regions of the lens, and by quantifying the differences in the phosphorylated proteins between HMC and ARC, we clarified the different pathogeneses in these two phenotypes.

We identified 522 phosphorylation sites in 164 phosphoproteins in this study. Previous studies have reported 73 phosphorylation sites and 32 phosphoproteins in normal and cataractous lenses, using immobilized metal affinity chromatography and nano-LC-coupled MS/MS [15]. *α*-Crystallin A and *α*-crystallin B are the most abundantly phosphorylated proteins in the porcine lens [16]. Our data show that besides these two crystallin proteins, beta A3, beta B1, beta B2, beta S, and gamma D crystallins were also phosphorylated at many peptide sites.

In this study, the number of phosphorylation sites was significantly greater in the lens proteins of the cortex than in those of the nuclear region, in both HMC and ARC. One possible explanation to this finding is that the lens epithelium cells immediately adjacent to areas of the cortex are metabolically relatively active and metabolites decrease from the lens cortex toward the lens nucleus. Protein enzymatically phosphorylated in the outer cortex could gradually dephosphorylate nonenzymatically in the metabolically inactive nucleus. Among the phosphoproteins in these groups, a high percentage of the differentially phosphorylated proteins were crystallins and lens structural proteins, including *β*-crystallin, *α*-crystallin, phakinin, and filensin.

*α*-Crystallin is a small heat shock protein that maintains the transparency of the lens. Phosphorylation is considered to change its chaperone activity by inducing a change in the protein's structure and altering the subunit exchange dynamics [17]. The phosphorylation of *α*-crystallin B has been shown to regulate the protein's activity in both lenticular and extralenticular tissues [5, 18–23]. The commonest functional modification sites in *α*-crystallin B are S19, S45, and S59. We detected these phosphopeptides in the lenses of patients with both HMC and ARC. When we compared the S19 site between these two groups, the level of phosphorylation was higher in the HMC-C than in the ARC-C. However, the pathological significance of these proteomic changes requires further analysis. There was a slight difference in the phosphorylation at S59 between the ARC-C and ARC-N and between the HMC-N and ARC-N. The phosphorylation of *α*-crystallin B at S59 is thought to be associated with actin nucleation and the migration of lens epithelial cells [18]. We detected no difference in the phosphorylation at S45 in any paired comparison of the four groups. Filensin and phakinin are two unique protein components of the lens fibers that assemble to form an intermediate filament, known as the beaded filament [24–27]. As previously reported, filensin and phakinin in the lens fiber cells are essential for maintaining the transparency of the lens [17]. As the lens fiber differentiates and with aging, these proteins become the targets of phosphorylation as a posttranslational modification [18]. It has also been reported that the phosphorylation of intermediate filament proteins plays an essential role in regulating the kinetics of these proteins, including their solubility, conversion, and the fiber structure [19–21].

In the HMC group, the phosphosites that differed strongly in their phosphorylation between the cortex and nucleus predominantly comprised enzymes involved in glutathione synthesis, including GSS and S-formylglutathione hydrolase. Our KEGG pathway analysis also showed that the largest proportion of phosphoproteins was associated with glutathione metabolism.

GSS and S-formylglutathione hydrolase catalyze key steps in glutathione synthesis. Glutathione is an essential antioxidant that protects the lens from oxidative damage [22]. The level of glutathione synthesis is lower in the cataractous lenses than in the lenses of emmetropic eyes but is lowest in myopic lenses [23]. It has also been demonstrated that eyes with high myopia are susceptible to oxidative damage and are associated with an increased incidence of nuclear cataract (with an adjusted odds ratio of 3.01) [5]. The lens typically exists in a low-oxygen environment [28, 29], and increased exposure to oxygen appears to cause cataract. Previous studies have shown that the degree of vitreous liquefaction is positively correlated with the level of nuclear opacity in the lens after adjustment for age [30]. As a possible mechanism, vitreous liquefaction increases the flow of fluid in the vitreous cavity and allows oxygen to flow from the retina to the lens. In patients with high myopia, vitreous liquefaction often occurs in the early stage of myopia and the severity of this complication increases as myopia worsens [31]. The glutathione content varies between different types of cataract. Subcapsular cataract, with an additional secondary nuclear cataract, shows a particularly rapid reduction in glutathione [32]. As a result, highly myopic eyes are more susceptible to oxidative damage than less myopic eyes, which leads to the formation of nuclear cataract. Consistent with this, our experimental data show that the number of phosphorylated glutathione synthase molecules was significantly higher in the cortex of the HMC lens than in the nucleus.

By comparing the catalogues of differentially phosphorylated protein in the cortical regions of HMC and ARC, results showed that the degree of phosphorylation of GSS and GSR, the key enzymes of glutathione synthesis, was higher in HMC than in ARC. However, the precise roles of GSR and GSS phosphorylation remain unclear.

In our comparison of the two lens tissues in HMC and ARC, the number of phosphopeptides was much higher in the HMC-N than in the ARC-N, which may be associated with the severity of nuclear cataract in patients with high myopia. Truscott [33] proposed that there is a barrier to the transport of metabolites within the lens. This barrier may increase the half-lives of reactive molecules, thus promoting the posttranslational modification of proteins in the nucleus, and may also prevent an adequate flux of antioxidants reaching the lens interior, thus allowing the oxidation of the nuclear components. Other authors have suggested that a common underlying mechanism in the pathology of cortical and nuclear cataract is the failure of the microcirculatory system to regulate the cell volume in the lens cortex or to deliver antioxidants to the lens nucleus [34]. Therefore, we suggest that the nuclear region of the cataractous lens may be a meaningful target region for the posttranslational modification of proteins. The HMC lens may be the best model to study the transfer of antioxidants to the nucleus through the barrier.

When we examined the glycolysis and metabolic pathways, which were enriched in differentially phosphorylated proteins in HMC and ARC, we found that the degree of phosphorylation of PGK1 was lower in HMC-C and HMC-N than in the ARC-C and ARC-N, respectively. A previous study showed that phosphorylation of PGK1 reduces its activity, thus reducing the glycolytic activity [35]. Glycolysis is the main source of energy generation in the unique low-oxygen environment of the eye. A reduction in energy metabolism impairs the activity of NA^+^-K^+^-ATPase in the lens, and the cascade reaction leads to an imbalance in lens homeostasis.

## 5. Conclusion

In summary, we analyzed the phosphoproteomes of the cortex and nucleus of HMC and ARC lenses, while considering the clinical features of the lenses. We found significant differences in the extent of protein phosphorylation and the types of proteins phosphorylated between different regions of the lens. Our results will be valuable for the future investigation of the molecular characteristics and pathological pathways underlying HMC and ARC.

## Figures and Tables

**Table 1:** Phosphosites differentially expressed in HMC-C vs HMC-N.

Protein names	Gene name	Accession	Score	Modified sequence	Position	FC	*p* value
Phosphosites exclusively detected in HMC-C							
Alcohol dehydrogenase 1A	ADH1A	P07327	106.43	_EIGADLVLQIS(ph)KESPQEIAR_	225	NaN	0.003
Alcohol dehydrogenase 1A	ADH1A	P07328	105.84	_S(ph)GGTLVLVGLGSEMTTVPLLHAAIR_	266	NaN	0.039
Aldehyde dehydrogenase family 1 member A3	ALDH1A3	P47895	88.19	_IFINNEWHES(ph)KSGK_	43	NaN	0.026
Filensin	BFSP1	Q12934	73.156	_S(ph)RS(ph)LPEKGPPK_	605	NaN	0.003
Filensin	BFSP1	Q12934	97.214	_VLEKS(ph)SYDCR_	288	NaN	0.034
Beta-crystallin B2	CRYBB2	P43320	94.45	_DSSDFGAPHPQVQS(ph)VRR_	186	NaN	0.010
Protein 4.1	EPB41	P11171	63.16	_KLS(ph)MYGVDLHK_	394	NaN	0.013
Glutathione reductase	GSR	P00390	74.12	_GHAAFT(ph)SDPKPTIEVSGK_	177	NaN	0.008
Phakinin	BFSP2	Q13515	102.6	_MDLESQIESLKEELGSLS(ph)R_	238	NaN	0.011
Galectin-1	LGALS1	P09382	74.12	_SFVLNLGKDS(ph)NNLCLHFNPR_	39	NaN	≤0.001
Leukotriene A-4 hydrolase	LTA4H	P09960	94.85	_CS(ph)VDFTR_	27	NaN	0.002
Mpv17-like protein 2	MPV17L2	Q567V2	106.13	_LLS(ph)AGQLLFQGR_	14	NaN	0.004
Neurofascin	NRCAM	O94856	78.342	_SGT(ph)LVIDFR_	98	NaN	0.001
Peroxiredoxin-6	PRDX6	P30041	50.364	_RVATPVDWKDGDS(ph)VMVLPTIPEEEAK_	186	NaN	0.005
cAMP-dependent protein kinase catalytic subunit alpha	PRKACA	P17612	68.108	_KGS(ph)EQESVK_	11	NaN	0.003
Ribose-phosphate diphosphokinase	PRPS2	A0A140VK41	53.865	_VAILVDDMADT(ph)CGTICHAADK_	228	NaN	≤0.001
Tryptophan-tRNA ligase, cytoplasmic	WARS	P23381	99.802	_KLS(ph)FDFQ_	467	NaN	≤0.001
Phosphosites exclusively detected in HMC-N							
Carbonic anhydrase 4	CA4	P22748	69.03	_EQT(ph)VSM(ox)KDNVR_	263	NaN	0.001
Carbonyl reductase (NADPH) 1	CBR1	P16152	75.462	_LFS(ph)GDVVLTAR_	30	NaN	≤0.001
Beta-crystallin A4	CRYBA4	P53673	60.564	_GEY(ph)PSWDAWGGNTAYPAER_	74	NaN	≤0.001
Glutathione synthetase	GSS	P48637	156.67	_QIEINTIS(ph)ASFGGLASR_	149	NaN	0.003
HSPC141	PHPT1	Q9P019	66.871	_(ac)AVADLALIPDVDIDS(ph)DGVFK_	16	NaN	0.001
Phosphosites with upregulation in HMC-C (selected)							
S-Formylglutathione hydrolase	ESD	P10768	117.37	_MSIFGHS(ph)MGGHGALICALK_	149	4.61	0.003
Glyceraldehyde-3-phosphate dehydrogenase	GAPDH	P04406	67.995	_IISNASCT(ph)TNCLAPLAK_	153	2.58	0.001
Filensin	BFSP1	Q12934	103.27	_LQLEAQFLQDDIS(ph)AAKDR_	170	2.57	0.037
Alpha-crystallin A chain	CRYAA	P02489	123.19	_QS(ph)LFRTVLDSGISEVR_	51	2.41	0.003
Fructose-bisphosphate aldolase	ALDOA	J3KPS3	80.229	_CQY(ph)VTEK_	208	2.39	0.023
Phosphosites with downregulation in HMC-C							
Gamma-crystallin C	CRYGC	P07315	125.77	_RGEYPDYQQWM(ox)GLS(ph)DSIR_	73	0.02	0.036
Fructose-bisphosphate aldolase	ALDOA	J3KPS3	132.4	_GILAADEST(ph)GSIAKR_	37	0.15	≤0.001
Beta-crystallin A3	CRYBA1	P05813	184.26	_VES(ph)GAWIGYEHTSFCGQQFILER_	70	0.45	0.031
Gamma-crystallin C	CRYGC	P07315	235.83	_VES(ph)GCWMLYERPNYQGQQYLLR_	40	0.48	0.029

**Table 2:** Phosphosites differentially expressed in ARC-C vs ARC-N.

Protein names	Gene name	Accession	Score	Modified sequence	Position	FC	*p* value
Phosphosites exclusively detected in ARC-C							
Actin, cytoplasmic 1	ACTA2	P60709	111.6	_GYS(ph)FTTTAER_	199	NaN	0.006
Alcohol dehydrogenase 1A	ADH1A	V9HW89	93.73	_SGGTLVLVGLGS(ph)EMTTVPLLHAAIR_	277	NaN	0.024
Alcohol dehydrogenase 1A	ADH1A	V9HW89	82.89	_EIGADLVLQISKES(ph)PQEIAR_	228	NaN	≤0.001
Alcohol dehydrogenase 1A	ADH1A	V9HW89	105.8	_S(ph)GGTLVLVGLGSEMTTVPLLHAAIR_	266	NaN	0.001
Cysteine protease	ATG4D	B4DZK0	64.3	_KYS(ph)IFTEKDEILSDVASR_	151	NaN	0.001
Alpha-crystallin A chain	CRYAA	P02489	142.5	_HFSPEDLT(ph)VK_	86	NaN	≤0.001
Beta-crystallin A2	CRYBA2	P53672	85.46	_LLS(ph)DCANVCER_	31	NaN	0.026
Beta-crystallin A4	CRYBA4	P53673	86.48	_GFQYVLECDHHS(ph)GDYK_	170	NaN	0.015
Beta-crystallin B1	CRYBB1	P53674	162.4	_WNTWSS(ph)SYR_	129	NaN	0.016
Quinone oxidoreductase	CRYZ	Q08257	98.77	_AGESVLVHGAS(ph)GGVGLAACQIAR_	158	NaN	≤0.001
Eukaryotic initiation factor 4A-II	EIF4A2	Q14240	137.9	_GYDVIAQAQS(ph)GTGK_	79	NaN	≤0.001
Protein 4.1	EPB41	P11171	99.02	_QAS(ph)ALIDRPAPHFER_	521	NaN	≤0.001
Glucose-6-phosphate isomerase (fragment)	GPI	A0A0A0MTS2	65.72	_ELQAAGKS(ph)PEDLER_	470	NaN	≤0.001
Heat shock 70 kDa protein 4	HSPA4	P34932	63.71	_AFS(ph)DPFVEAEK_	76	NaN	≤0.001
Glutathione synthetase	GSS	P48637	68.94	_DGY(ph)MPRQYSLQNWEAR_	270	NaN	0.003
Phakinin	BFSP2	Q13515	110.9	_AAEEEINS(ph)LYK_	208	NaN	≤0.001
Phakinin	BFSP2	Q13515	58.69	_VHALEQVSQELET(ph)QLR_	134	NaN	0.006
Lactase-like protein	LCT	Q6UWM7	86.29	_S(ph)AEQGLEM(ox)SR_	311	NaN	≤0.001
L-Lactate dehydrogenase A chain	LDHC	P00338	90.61	_S(ph)ADTLWGIQK_	319	NaN	0.028
Neurofascin	NRCAM	O94856	78.34	_SGT(ph)LVIDFR_	98	NaN	≤0.001
Protein kinase C and casein kinase substrate in neurons 3	PACSIN3	D3DQR0	92.54	_LKEVEAS(ph)K_	153	NaN	0.001
Peroxisome biogenesis factor 10, isoform CRA_b	PEX10	A0A024R0A4	87.26	_RAS(ph)LEER_	281	NaN	0.006
Phosphoglycerate mutase 1	PGAM1	P18669	56.72	_FSGWYDADLS(ph)PAGHEEAKR_	31	NaN	0.016
Plectin	PLEC	Q15149	93.16	_LS(ph)FSGLR_	3441	NaN	≤0.001
Plectin	PLEC	Q15149	69.72	_KAS(ph)DSELER_	2039	NaN	≤0.001
Plectin	PLEC	Q15149	78.65	_KES(ph)YSALMR_	794	NaN	0.007
cAMP-dependent protein kinase catalytic subunit alpha	PRKACA	P17612	68.11	_KGS(ph)EQESVK_	11	NaN	≤0.001
Ribose-phosphate diphosphokinase	PRPS2	A0A140VK41	53.87	_VAILVDDMADT(ph)CGTICHAADK_	228	NaN	≤0.001
Glycogen phosphorylase	PYGB	P06737	61.64	_RMS(ph)LIEEEGSKR_	430	NaN	0.002
SEC14-like protein 2	SEC14L2	O76054	122.5	_VGDLS(ph)PR_	9	NaN	≤0.001
Tryptophan-tRNA ligase, cytoplasmic	WARS	P23381	99.8	_KLS(ph)FDFQ_	467	NaN	0.002
Synaptobrevin homolog YKT6	YKT6	O15498	53.03	_IDWPVGS(ph)PATIHYPALDGHLSR_	114	NaN	0.010
14-3-3 protein	YWHAH	Q04917	51.15	_KNS(ph)VVEASEAAYK_	145	NaN	≤0.001
Phosphosites exclusively detected in ARC-N							
Nucleoside triphosphate pyrophosphatase	ASMT	O95671	80.98	_VVLASAS(ph)PR_	21	NaN	≤0.001
Alpha-crystallin B chain	CRYAA	P02511	127	_LFDQFFGEHLLESDLFPTSTSLS(ph)PFYLRPPSFLR_	45	NaN	0.010
Crystallin gamma B	CRYGB	A0A0U3BWM0	73.78	_GQMSELT(ph)DDCLSVQDR_	107	NaN	0.004
3-Hydroxyanthranilate 3,4-dioxygenase	HAAO	P46952	78.43	_RLS(ph)LAPDDSLLVLAGTSYAWER_	247	NaN	0.007
Phosphosites with upregulation in ARC-C (selected)							
Beta-crystallin A3	CRYBA1	P05813	69.26	_ITIY(ph)DQENFQGK_	36	####	≤0.001
Alpha-crystallin B chain	CRYAA	P02511	177.9	_RPFFPFHSPS(ph)R_	21	####	0.049
Phakinin	BFSP2	Q13515	174.6	_SS(ph)SSLES(ph)PPASR_	38	####	≤0.001
Alpha-crystallin B chain	CRYAA	P02511	151.2	_LEKDRFS(ph)VNLDVK_	76	####	0.001
Filensin	BFSP1	Q12934	173.6	_VRS(ph)PKEPETPTELYTK_	454	####	0.009
Phosphosites with downregulation in ARC-C							
Beta-crystallin A3	CRYBA1	P05813	252.2	_RMEFTS(ph)SCPNVSER_	50	0.26	0.005
Gamma-crystallin D	CRYGD	P07320	151.2	_RGDYADHQQWMGLS(ph)DSVR_	73	0.27	0.009
Coactosin-like protein	COTL1	Q14019	70.09	_FTTGDAMS(ph)KR_	71	0.45	≤0.001
Beta-crystallin A3	CRYBA1	P05813	123.4	_WDAWS(ph)GSNAYHIER_	100	0.50	0.049

**Table 3:** Phosphosites differentially expressed in HMC-C vs ARC-C.

Protein names	Gene name	Accession	Score	Modified sequence	Position	FC	*p* value
Phosphosites exclusively detected in HMC-C							
Fructose-bisphosphate aldolase	ALDOA	J3KPS3	80.229	_CQY(ph)VTEK_	208	NaN	≤0.001
N-Acetylserotonin O-methyltransferase-like protein	ASMT	O95671	80.979	_VVLASAS(ph)PR_	21	NaN	≤0.001
Alpha-crystallin A chain	CRYAA	P02489	193.97	_YRLPSNVDQS(ph)ALSCSLSADGMLTFCGPK_	127	NaN	0.047
Beta-crystallin B1	CRYBB1	P53674	163.34	_WNTWS(ph)SSYR_	128	NaN	0.014
Beta-crystallin B3	CRYBB3	P26998	69.122	_CELS(ph)AECPSLTDSLLEK_	42	NaN	≤0.001
Gamma-crystallin C	CRYGC	P07315	111.12	_SCCLIPQT(ph)VSHR_	85	NaN	0.003
Glutathione reductase, mitochondrial	GSR	P00390	74.12	_GHAAFT(ph)SDPKPTIEVSGK_	177	NaN	0.008
Phakinin	BFSP2	Q13515	116.43	_S(ph)SSS(ph)LESPPASR_	35	NaN	≤0.001
Galectin-1	LGALS1	P09382	74.12	_SFVLNLGKDS(ph)NNLCLHFNPR_	39	NaN	≤0.001
Mpv17-like protein 2	MPV17L2	Q567V2	106.13	_LLS(ph)AGQLLFQGR_	14	NaN	0.004
Ubiquitin C variant (fragment)	UBC	Q59EM9	122.44	_TIT(ph)LEVEPSDTIENVK_	30	NaN	≤0.001
14-3-3 protein zeta/delta (fragment)	YWHAB	E7EX29	55.885	_DICNDVLS(ph)LLEK_	99	NaN	0.003
Phosphosites exclusively detected in ARC-C							
Alcohol dehydrogenase 1A	ADH1A	V9HW89	124.19	_AMGAAQVVVTDLSATRLS(ph)K_	211	NaN	0.048
Alcohol dehydrogenase 2A	ADH1A	V9HW89	82.885	_EIGADLVLQISKES(ph)PQEIAR_	228	NaN	≤0.001
Carbonyl reductase (NADPH) 1	CBR1	P16152	75.462	_LFS(ph)GDVVLTAR_	30	NaN	≤0.001
Alpha-crystallin B chain	CRYAA	P02511	152.88	_IPADVDPLTITS(ph)SLSSDGVLTVNGPR_	135	NaN	≤0.001
Beta-crystallin B1	CRYBB1	P53674	162.38	_WNTWSS(ph)SYR_	129	NaN	0.016
Protein 4.1	EPB41	P11171	99.021	_QAS(ph)ALIDRPAPHFER_	521	NaN	≤0.001
Glucose-6-phosphate isomerase (fragment)	GPI	A0A0A0MTS2	65.716	_ELQAAGKS(ph)PEDLER_	470	NaN	≤0.001
Heat shock 70 kDa protein 4	HSPA4	P34932	63.709	_AFS(ph)DPFVEAEK_	76	NaN	≤0.001
Phakinin	BFSP2	Q13515	151.44	_S(ph)S(ph)SSLESPPASR_	32	NaN	≤0.001
Phakinin	BFSP2	Q13515	58.693	_VHALEQVSQELET(ph)QLR_	134	NaN	0.006
L-Lactate dehydrogenase A chain	LDHC	P00338	90.614	_S(ph)ADTLWGIQK_	319	NaN	0.028
Phosphoglycerate mutase 1	PGAM1	P18669	56.72	_FSGWYDADLS(ph)PAGHEEAKR_	31	NaN	0.016
Synaptobrevin homolog YKT6	YKT6	O15498	53.033	_IDWPVGS(ph)PATIHYPALDGHLSR_	114	NaN	0.010
14-3-3 protein eta	YWHAH	Q04917	51.147	_KNS(ph)VVEASEAAYK_	145	NaN	≤0.001
Phosphosites with upregulation in HMC-C (selected)							
Gamma-crystallin D	CRYGD	P07320	130.01	_HYECSSDHPNLQPY(ph)LSR_	29	29.25	0.006
Alpha-crystallin B chain	CRYAA	P02511	184.11	_RPFFPFHS(ph)PSR_	19	11.99	0.003
Alpha-crystallin A chain	CRYAA	P02489	155.17	_T(ph)LGPFYPSR_	13	5.08	≤0.001
Quinone oxidoreductase PIG3	TP53I3	Q53FA7	141.54	_RGS(ph)LITSLLR_	260	3.92	≤0.001
Alpha-crystallin A chain	CRYAA	P02489	123.19	_QS(ph)LFRTVLDSGISEVR_	51	2.77	0.013
Phosphosites with downregulation in HMC-C (selected)							
Phakinin	BFSP2	Q13515	174.62	_SS(ph)SSLES(ph)PPASR_	38	0.05	0.048
Filensin	BFSP1	Q12934	173.55	_VRS(ph)PKEPETPTELYTK_	454	0.20	0.026
Filensin	BFSP1	Q12934	173.55	_VRS(ph)PKEPET(ph)PTELYTK_	460	0.26	≤0.001
Retinal dehydrogenase 1	ALDH1A1	P00352	78.921	_YILGNPLT(ph)PGVTQGPQIDKEQYDK_	337	0.28	≤0.001
Brain acid soluble protein 1	BASP1	P80723	127.95	_AEGAATEEEGT(ph)PK_	36	0.30	0.008

**Table 4:** Phosphosites differentially expressed in HMC-N vs ARC-N.

Protein names	Gene name	Accession	Score	Modified sequence	Position	FC	*p* value
Phosphosites exclusively detected in HMC-N							
Retinal dehydrogenase 1	ALDH1A1	P00352	78.92	_YILGNPLT(ph)PGVTQGPQIDKEQYDK_	337	NaN	0.019
Fructose-bisphosphate aldolase	ALDOA	J3KPS3	92.46	_RTVPPAVTGITFLS(ph)GGQSEEEASINLNAINK_	276	NaN	0.008
Fructose-bisphosphate aldolase	ALDOA	J3KPS3	80.23	_CQY(ph)VTEK_	208	NaN	≤0.001
Cysteine protease	ATG4D	B4DZK0	64.3	_KYS(ph)IFTEKDEILSDVASR_	151	NaN	0.014
Carbonic anhydrase	CA2	P00918	61.44	_EPIS(ph)VSSEQVLK_	216	NaN	0.001
Alpha-crystallin A chain	CRYAA	P02489	142.5	_HFSPEDLT(ph)VK_	86	NaN	≤0.001
Beta-crystallin A4	CRYBA4	P53673	86.48	_GFQYVLECDHHS(ph)GDYK_	170	NaN	≤0.001
Beta-crystallin A4	CRYBA4	P53673	60.56	_GEY(ph)PSWDAWGGNTAYPAER_	74	NaN	≤0.001
Beta-crystallin B3	CRYBB3	P26998	69.12	_CELS(ph)AECPSLTDSLLEK_	42	NaN	≤0.001
Quinone oxidoreductase	CRYZ	Q08257	98.77	_AGESVLVHGAS(ph)GGVGLAACQIAR_	158	NaN	0.011
Eukaryotic initiation factor 4A-II	EIF4A2	Q14240	137.9	_GYDVIAQAQS(ph)GTGK_	79	NaN	0.001
S-Formylglutathione hydrolase	ESD	P10768	117.4	_MSIFGHS(ph)MGGHGALICALK_	149	NaN	≤0.001
Glyceraldehyde-3-phosphate dehydrogenase	GAPDH	P04406	68	_IISNASCT(ph)TNCLAPLAK_	153	NaN	0.012
Glutathione synthetase	GSS	P48637	156.7	_QIEINTIS(ph)ASFGGLASR_	149	NaN	0.003
Inosine-5′-monophosphate dehydrogenase	IMPDH1	Q5H9Q6	121.6	_LVGIVT(ph)SR_	234	NaN	0.009
Phakinin	BFSP2	Q13515	116.4	_S(ph)SSS(ph)LESPPASR_	35	NaN	0.015
Protein kinase C and casein kinase substrate in neurons 3	PACSIN3	D3DQR0	92.54	_LKEVEAS(ph)K_	153	NaN	0.032
Peroxisome biogenesis factor 10, isoform CRA_b	PEX10	A0A024R0A4	87.26	_RAS(ph)LEER_	281	NaN	≤0.001
HSPC141	PHPT1	Q9P019	66.87	_(ac)AVADLALIPDVDIDS(ph)DGVFK_	16	NaN	0.001
Plectin	PLEC	Q15149	93.16	_LS(ph)FSGLR_	3441	NaN	0.019
Plectin	PLEC	Q15149	69.72	_KAS(ph)DSELER_	2039	NaN	≤0.001
Glycogen phosphorylase, liver form	PYGB	P06737	61.64	_RMS(ph)LIEEEGSKR_	430	NaN	0.036
SEC14-like protein 2	SEC14L2	O76054	122.5	_VGDLS(ph)PR_	9	NaN	≤0.001
Ubiquitin C variant (fragment)	UBC	Q59EM9	122.4	_TIT(ph)LEVEPSDTIENVK_	30	NaN	0.009
14-3-3 protein zeta/delta (fragment)	YWHAB	E7EX29	55.89	_DICNDVLS(ph)LLEK_	99	NaN	≤0.001
Phosphosites exclusively detected in ARC-N							
Alpha-crystallin B chain	CRYAA	P02511	127	_LFDQFFGEHLLESDLFPTSTSLS(ph)PFYLRPPSFLR_	2	NaN	0.010
Beta-crystallin A3	CRYBA1	P05813	103.2	_WDAWSGSNAY(ph)HIER_	174	NaN	0.009
3-Hydroxyanthranilate 3,4-dioxygenase	HAAO	P46952	78.43	_RLS(ph)LAPDDSLLVLAGTSYAWER_	160	NaN	0.007
Protein NDRG1	NDRG1	Q92597	96.54	_S(ph)REMQDVDLAEVKPLVEK_	56	NaN	≤0.001
Phosphosites with upregulation in HMC-N (selected)							
Alpha-crystallin B chain	CRYAA	P02511	177.9	_RPFFPFHSPS(ph)R_	21	####	0.020
Alpha-crystallin B chain	CRYAA	P02511	84.3	_EEKPAVT(ph)AAPK_	170	####	0.000
Alpha-crystallin B chain	CRYAA	P02511	151.2	_LEKDRFS(ph)VNLDVK_	76	####	0.004
Beta-crystallin B1	CRYBB1	P53674	154.7	_QWHLEGSFPVLAT(ph)EPPK_	248	####	≤0.001
Filensin	BFSP1	Q12934	58.89	_KEQYEHADEAS(ph)R_	22	8.20	0.002
Phosphosites with downregulation in HMC-N							
Phosphoglycerate kinase 1	PGK1	P00558	69.38	_AHS(ph)S(ph)MVGVNLPQK_	174	0.38	0.005
Carbonyl reductase (NADPH) 1	CBR1	P16152	228.2	_FRS(ph)ETITEEELVGLMNK_	160	0.47	0.008
Carbonyl reductase (NADPH) 1	CBR1	P16152	207.6	_GQAAVQQLQAEGLS(ph)PR_	56	0.47	0.019
Beta-crystallin S	CRYGS	P22914	190.8	_KPIDWGAASPAVQS(ph)FRR_	172	0.49	0.016

## Data Availability

The data supporting the findings of this study are available within the article and its supplementary materials.

## Supplementary Materials

Supplementary 1 Table S1. The clinical information for the lens samples.

Supplementary 2 Table S2. Phosphopeptide annotation in HMC and ARC lenses.
