# Supplementary material for: Quantitative Phosphoproteomic Comparison of Lens Proteins in Highly Myopic Cataract and Age-Related Cataract
Source: Biomed Res Int. 2021 May 10;2021:6668845. doi: 10.1155/2021/6668845 (PMC8130905; doi:10.1155/2021/6668845)

Group	Age	LOCS III		AL(mm)
		C	N	
Highly myopic cataract group	66.67±2.08	2.93±0.12	3.50±0.50	30.17±1.02
Age-related cataract group	67.33±1.53	3.33±0.58	3.33±0.58	23.71±1.06
p -value	0.68	0.305	0.725	0.002*

LOCS=Lens Opacities Classification System

AL=Axial length

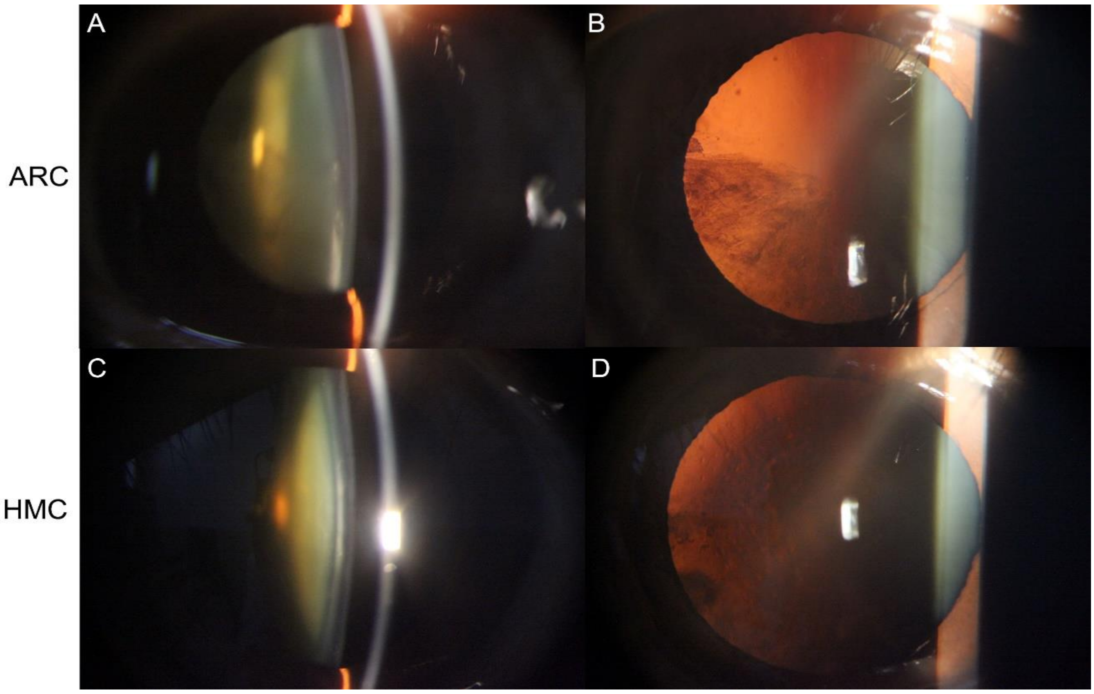

Supplement: Supplementary 1 — Table S1. The clinical information for the lens samples. [file 6668845.f1.pdf]
